# Functional Polymorphism of IL-1 Alpha and Its Potential Role in Obesity in Humans and Mice

**DOI:** 10.1371/journal.pone.0029524

**Published:** 2011-12-27

**Authors:** Jae-Young Um, Hong-Kun Rim, Su-Jin Kim, Hye-Lin Kim, Seung-Heon Hong

**Affiliations:** 1 Department of Pharmacology, College of Oriental Medicine, Institute of Oriental Medicine, Kyung Hee University, Dongdaemun-gu, Seoul, Republic of Korea; 2 Department of Cosmeceutical Science, Daegu Hanny University, Yugok-dong, Kyungsan, Republic of Korea; 3 Department of Oriental Pharmacy, College of Pharmacy, Wonkwang University, Iksan, Jeonbuk, Republic of Korea; I2MC INSERM UMR U1048, France

## Abstract

Proinflammatory cytokines secreted from adipose tissue contribute to the morbidity associated with obesity. IL-1α is one of the proinflammatory cytokines; however, it has not been clarified whether IL-1α may also cause obesity. In this study, we investigated whether polymorphisms in IL-1α contribute to human obesity. A total of 260 obese subjects were genotyped for IL-1α C-889T (rs1800587) and IL-1α G+4845T (rs17561). Analyses of genotype distributions revealed that both IL-1α polymorphisms C-889T (rs1800587) and G+4845T (rs17561) were associated with an increase in body mass index in obese healthy women. In addition, the effect of rs1800587 on the transcriptional activity of IL-1α was explored in pre-adipocyte 3T3-L1 cells. Significant difference was found between the rs1800587 polymorphism in the regulatory region of the IL-1α gene and transcriptional activity. We extended these observations in vivo to a high-fat diet-induced obese mouse model and in vitro to pre-adipocyte 3T3-L1 cells. IL-1α levels were dramatically augmented in obese mice, and triglyceride was increased 12 hours after IL-1α injection. Taken together, IL-1α treatment regulated the differentiation of preadipocytes. IL-1α C-889T (rs1800587) is a functional polymorphism of IL-1α associated with obesity. IL-1α may have a critical function in the development of obesity.

## Introduction

Worldwide, more than one billion adults are overweight or obese, and there in no sign that the rapid increase in obesity seen over the past two decades is abating. Obesity is recognized as a major risk factor for insulin resistance, and both of these conditions predict the development of type 2 diabetes mellitus and cardiovascular disease [1]. One emerging feature of obesity is the linkage between obesity and chronic, low-grade inflammation characterized by increased cytokine and chemokine production and acute-phase inflammatory signaling in adipose tissue [2], [3]. In fact, inflammatory markers, such as C-reactive protein (CRP) and interleukin (IL)-6, are increased in obese individuals compared with lean subjects, although not to the same extent observed in classic inflammatory conditions [4], [5].

White adipose tissue (WAT) is characterized by the ability to produce and release a variety of proinflammatory adipokines such as leptin, IL-1β, IL-6, IL-8, tumor necrosis factor (TNF)-α, monocyte chemoattractant protein-1, and macrophage migration inhibitory factor, all of which have been linked to insulin resistance [3]. IL-1 is also one of the major proinflammatory cytokines. It induces fever, synthesis of hepatic acute phase proteins, and the release of neutrophils as a mediator of acute inflammatory responses together with some other cytokines [6]. IL-1 is produced and secreted by a variety of cells including macrophages/monocytes, endothelial cells, vascular smooth muscle cells, and hepatocytes [7]–[9]. Dinarello et al. [10] have reported that the production of IL-1 is increased in diabetic patients as well as in patients with rheumatoid arthritis or with cancers, suggesting that IL-1 may play a role in the pathogenesis of diabetes mellitus. Di Renzo et al. [11] demonstrated higher levels of IL-1 in obese subjects. Raymond et al. [12] also reported that IL-1 α production by cultured peripheral blood mononuclear cells from the obese group was significantly elevated in comparison to the control group. However, it remains unclear whether or how IL-1 affects obesity.

The IL-1 gene family consists of two major agonistic molecules, namely, IL-1α and IL-1β, and one antagonistic cytokine, the IL-1 receptor antagonist (IL-1Ra) [7]. Both IL-1α and IL-1β are produced by lymphocytes or monocytes in the loci of inflammation. Only a few studies have examined the role of IL-1α as a mediator for cellular insulin resistance [6] in sharp contrast to a number of reports on IL-1β [13], [14].

Most of the genes coding for the IL-1 family of proteins and clustered on the 2q12-q21 locus (IL-1α, IL-1β, and IL-1Ra) are polymorphic in multiple loci [15]. A single nucleotide polymorphism (SNP) of the IL-1α gene was located at position -889 in the 5′-flanking region and the other was found at position +4845. Dominici *et al.* [16] reported that lipopolysaccharide-stimulated mononuclear cells from rs1800587 TT carriers showed an increase in the production of protein. They also found that the IL-1α promoter activity is higher in those individuals showing the TT –889 genotype over the CC –889 genotype. However Kawaguchi *et al.* [17] reported that significant differences in luciferase activity were not detected between C and T at –889 in systemic sclerosis fibroblasts, while the SNP at +4845 contributed to the processing of pre-IL1α in human skin fibroblasts.

Obesity is defined by body mass index (BMI) and further evaluated in terms of fat distribution via the waist-to-hip ratio (WHR) and total cardiovascular risk factors [18]. BMI is closely related to both percentage of body fat (PBF) and total body fat. Here, we investigated whether polymorphisms in IL-1α contribute to BMI in humans. We also hypothesized that the increased/decreased expression of IL-1α as a consequence of IL-1α C-889T (rs1800587) and IL-1α G+4845T (rs17561) polymorphisms could be a factor in obesity. We further extended this observation in vivo to a high-fat diet (HFD)-induced obese mouse model and in vitro to pre-adipocyte 3T3-L1 cells.

## Materials and Methods

### Ethics Statement

Written informed consent was obtained from all participants. The study was approved by the ethical committee of the Kyung Hee Oriental Medical Hospital. Some of the patients have been the subject of earlier report [19].

Mouse care and experimental procedures were performed under approval of the Kyung Hee University Institutional Animal Care and Use Committee (approval ID KHUASP(SE)-09-031) and were in compliance with the national and international laws for the protection and welfare of animals.

### Subjects

The subjects were recruited consecutively from an obesity clinic at a hospital from January 2000 to December 2003 into an ongoing project to investigate candidate genes for obesity among the Korean population. All were nonsmokers and had no evidence of cancer, liver, renal, hematological disease or other metabolic disorders other than obesity. A total of 260 women met all of the study criteria and were enrolled in the study. Ages and BMI ranged between 18 and 62 years and between 19.2 and 54.7 kg/m^2^, respectively. To obtain a better separation between phenotypes, the participants were divided into four BMI groups according to WHO definitions with minor modifications: lean (BMI <25 kg/m^2^), overweight I (BMI 25–26 kg/m^2^), overweight II (BMI 27–29 kg/m^2^) and obese (BMI ≥30 kg/m^2^).

### Phenotype measurements

Height (in cm) and weight (in kg) were measured to calculate the BMI as weight (kg)/height (m) squared. Waist circumference (measured at the narrowest point superior to the hip) was divided by the circumference of the hip (measured at its greatest gluteal protuberance) to obtain the WHR. Fat mass was determined by dual-energy X-ray absorptiometry.

### Genotype Determination

Genomic DNA was extracted from whole blood using Exgene Blood SV kit (GeneAll Biotechnology, Seoul, Republic of Korea). Genotyping for the IL-1α C–889T (rs1800587) was conducted essentially according to previous studies [20] with small modifications. The IL-1α G+4845T (rs17561) was amplified using the primers 5′-ATG GTT TTA GAA ATC ATC AAG CCT AGG GCA-3′ (forward) and 5′-ATT GAA AGG AGG GGA GGA TGA CAG AAA TGT-3′(reverse). The PCR products were digested with *Fnu* 4H1 (New England Biolabs, Ipswich, MA) [21].

### Reagents for Animal Experiments

ELISA capture and detection antibody and recombinant (standard) were purchased from R&D Systems (Minneapolis, Minnesota, USA). Dulbecco's Modified Eagle's Medium (DMEM), lipopolysaccharide (LPS), 3-isobutyl-1-methylxanthine (IBMX), insulin, and dexamethasone acetate were purchased from Sigma (St. Louis, MO). Western antibodies were obtained from Santa Cruz Biotechnology, Inc. (Santacruz, CA). Fetal bovine serum (FBS) and other tissue culture reagents were purchased from Gibco BRL (Grand Island, NY).

### IL-1α CC/TT Construct and Transfections

Luciferase reporter plasmid pGL3-Basic (Promega) was used in a reporter gene assay to examine IL-1α promoter activity. A fragment of 1,432 bp covering the IL-1α 5′-flanking sequence (nucleotide −1351 to +81) was amplified from genomic DNA containing either a C or T nucleotide at position –889 using the following primers: forward 5′-*GGG GT*A CCA GGT CTA TGA CCA GGA GAA TT-3′ and reverse 5′-*CCA AG*C TTT AAT AGC CAG AGA TGT GAG G-3′ (the *Kpn*I and *Hind*III restriction sites are italicized). The 1,432 bp products were then inserted (*Kpn*I and *Hind*III sites) into the pGL3-Basic vector [16]. All constructs were fully sequenced (LabFrontier Co. Ltd., Ehwa University, Seoul, Republic of Korea) before use in transfection experiments. The pGL3 constructs were transiently transfected into the 3T3-L1 cell line (American Type Culture Collection). Cells were transiently transfected using cationic liposomes (Transfast™ Transfection Reagent, Promega). The cells were co-transfected with 2 µg of construct DNA and 1.2 µg of the β-galactosidase plasmid DNA (pSV-β-gal, Promega). For each cell extract, luciferase activity assays were performed in triplicate using the standard protocol (Luciferase Assay System, Promega), and light was measured with a luminometer (VICTOR Light, Perkin Elmer).

### Animals

Four-week-old C57BL/6J male mice were purchased from the Daehan Biolink Co., (Daejeon, South Korea). The animals were maintained at the college of Oriental Medicine, Kyung Hee University.

### IL-1α Administration

Eight-week-old C57BL/6J male mice were given an intraperitoneal (IP) injection of recombinant IL-1α. IL-1α was diluted to the desired concentration with normal saline. IL-1α (10 µg/kg body weight) or vehicle (normal saline) was administered IP respectively. Livers and blood samples were collected 0, 4, 12, and 24 hours after IL-1α or vehicle injection and immediately frozen in a deep freezer.

### Lipid Analysis

Serum was separated immediately after blood sampling by centrifugation at 10,000× g for 15 min. Lipids were extracted from the livers by the method of Folch et al. [22]. Levels of total cholesterol, triglyceride, and LDL cholesterol were determined using the colorimetric enzymatic method, using an autoanalyzer (Hitachi 747; Hitachi, Japan).

### Cell Culture and Treatment

3T3-L1 fibroblasts were grown in DMEM plus 10% calf serum and plated for final differentiation in DMEM plus 10% FBS. Two days after reaching confluence, the medium was changed to the differentiation medium containing IBMX (0.5 mM), dexamethasone (0.25 µM), and insulin (10 µg/ml). After 2 days, the cell culture medium was changed to DMEM containing 10 µg/ml insulin and 10% FBS. The medium was replaced again with fresh DMEM containing 10% FBS after 2 days. The cells were treated with various concentrations of recombinant IL-1α during the differentiation induction phase (from day 0 to day 8). Cells and medium were collected 8 days after the initiation of differentiation.

### Oil Red O Staining

The 3T3-L1 cells were washed twice with phosphate-buffered saline (PBS) and were then fixed in 10% formaldehyde in PBS for 1 h. After washing with 60% isopropanol, the cells were stained with Oil Red O solution for 30 min at room temperature. The cells were washed with water four times to remove the unbound dye and were then photographed. To determine the extent of adipose conversion, 1 ml of isopropanol was added to the stained culture plates. The extracted dye was collected, and its optical density was measured at 500 nm using an automated microplate ELISA reader.

### RNA Isolation and Real-time PCR

Total RNA was extracted from culture cell and adipose tissue samples using a GeneAll^R^ RiboEx Total RNA extraction kit (GeneAll Biotechnology, Seoul, Republic of Korea) and QIAzol RNA extraction kit (Qiagen, USA). Real-time PCR was run on an Applied Biosystems StepOne Thermal Cycler with a 48-well reaction plate. A probe for each gene was designed with the Primer Express 3.0 program (Applied Biosystems, Foster City, CA, USA) with a 5′-FAM and 3′-TAMRA modification in addition to forward and reverse primers. Data were analyzed with StepOne software 2.0 (Applied Biosystems, Foster City, CA, USA).

### Adipokine Assay

The amounts of IL-1α, IL-6, and adiponectin were measured using standard kits (R&D systems, Inc., Minneapolis, MN, USA). The ELISA was devised by coating 96-well plates of murine monoclonal antibody with specificity for IL-1α, IL-6, and adiponectin. Color development was measured at 405 nm using an automated microplate ELISA reader.

### Statistical Analysis

Statistical analyses were performed by one-way analysis of variance (ANOVA) with Tukey, and Duncan post-hoc test to express the difference between groups. Data are presented as the mean ± standard deviations (SD). The significance of the genotype differences between groups was tested by χ^2^. Odds ratios were determined by the Mantel-Haenszel method. All statistical analyses were performed using SPSS v13 (SPSS Inc, Chicago, Illinois, USA). *P*<0.05 was considered statistically significant.

## Results

### Clinical Characteristics of Human Subjects

Anthropometric, biochemical, and clinical characteristics of subjects are summarized in Table 1. As expected, total cholesterol, triglyceride, fat mass, PBF, and WHR increased in proportion to BMI values.

**Table 1:** Characteristics of female subjects (*n* = 260).

	BMI (kg/m^2^)			
	<25	25–26	27–29	≥30
Weight* (kg)	61.5±13.8	64.4±4.6	71.0±5.9	84.8±13.4
Height* (cm)	161.1±5.1	158.6±5.9	158.5±5.6	161.2±5.6
Total cholesterol* (mg/dl)	168.2±26.9	181.3±40.3	179.1±34.7	189.8±40.7
Triglyceride* (mg/dl)	79.8±35.2	105.6±99.5	109.2±70.7	131.8±116.7
Fat mass* (kg)	20.1±2.1	23.3±2.5	26.6±3.2	36.2±8.2
PBF* (%)	33.6±2.3	36.1±2.9	36.9±5.2	41.9±4.4
WHR*	0.85±0.03	0.89±0.02	0.92±0.04	0.99±0.07

A total of 260 female subjects (mean age = 29.3±10.19) were included. Values are means ± SD. Abbreviations used: BMI, body mass index; PBF, percentage of body fat; WHR, ratio of waist-to-hip circumstance.

*Oneway ANOVA, *P*<0.05.

### Association between IL-1 α Polymorphism and BMI in Human Obese Patients

The genotype frequencies of IL-1α C-889T (rs1800587) and IL-1α G+4845T (rs17561) were in the Hardy-Weinberg equilibrium. To evaluate the association between the IL-1α polymorphisms and BMI further, subjects were grouped according to their BMI range. The distributions of CC, CT, and TT genotypes of IL-1α C-889T (rs1800587) and the distributions of GG, GT, and TT genotypes of IL-1α G+4845T (rs17561) in the study subjects are shown in Table 2. The frequency of rs1800587 CT+TT genotypes was lower in a subgroup with BMI 27–29 kg/m^2^ than in a subgroup with a BMI of less than 25 kg/m^2^ (odds ratio, OR = 0.183, confidence interval, CI = 0.07–0.50, *P* = 0.001). In addition, the GT+TT genotype frequency of rs17561 polymorphism was also lower in the BMI 27–29 kg/m^2^ subgroup than in lean women (BMI <25 kg/m^2^) (OR = 0.211, CI = 0.08–0.55, *P* = 0.001). To further investigate the significant associations between T carriers of IL-1α rs1800587 and rs17561 with BMI, haplotype analysis was also performed. The frequency of carriers with one or two copies of the rs1800587:rs17561 T:T haplotype was significantly lower in a subgroup with BMI 27–29 kg/m^2^ than in lean women (BMI <25 kg/m^2^) (OR = 0.217, CI = 0.08–0.56, *P* = 0.002). In addition, we evaluated whether the IL-1α polymorphisms affect other parameters such as WHR, or PBF. As a result, WHR was associated with the IL-1α polymorphisms (rs1800587 and rs17561), but not PBF (Table S1 and S2). The frequency of carriers with one or two copies of the rs1800587:rs17561 T:T haplotype was significantly lower in a subgroup with WHR greater than or equal to 0.91 than in women with WHR less than 0.90 (*P* = 0.006) (Table S2).

**Table 2:** Frequencies for IL-1α genotypes according to BMI in females (*n* = 260).

		BMI (kg/m^2^)				*P* a
		<25,n (%)	25–26,n (%)	27–29,n (%)	≥30,n (%)	
IL-1α C-889T(rs1800587)	CC	39 (68.4)	47 (81.0)	71 (92.2)	55 (80.9)	0.003
	CT	18 (31.6)	9 (15.5)	6 (7.8)	13 (19.1)	
	TT	0(0)	2 (3.4)	0 (0)	0 (0)	
IL-1α G+4845T(rs17561)	GG	38 (67.9)	47 (81.0)	70 (90.9)	56 (81.2)	0.004
	GT	18 (32.1)	9 (15.5)	7 (9.1)	13 (18.8)	
	TT	0 (0)	2 (3.4)	0 (0)	0 (0)	
rs1800587: rs17561 haplotype						
o copies T:T		39 (68.4)	47 (81.0)	70 (90.9)	55 (80.9)	0.013
1 or 2 copies T:T		18 (31.6)	11 (19.0)	7 (9.1)^b^	13 (19.1)	

BMI, body mass index; OR, odds ratio; CI, confidence interval.

a By χ^2^ test (two-sided);

b By Mantel-Haenszel method; women with BMI <25 kg/m^2^ were the reference in this analysis: *P* = 0.002, OR = 0.217, CI 0.08–0.56.

### IL-1α Levels in Human Obese Patients

To investigate whether the observed association has any physiological (pathophysiological) relevance, plasma IL-1α and CRP concentrations were measured in the four subgroups. From the 260 subjects, a subset of 30 subjects was recalled for plasma IL-1α and CRP measurements (BMI <25 kg/m^2^, *n* = 11; BMI 25–26 kg/m^2^, *n* = 2; BMI 27–29 kg/m^2^, *n* = 10; BMI ≥30 kg/m^2^, *n* = 7). An increase was found for circulating IL-1α concentrations in a subgroup with a BMI of 27–29 kg/m^2^ (8.39±1.10 pg/ml) compared to a subgroup with a BMI lower than 25 kg/m^2^ (7.65±0.55 pg/ml), although the statistical significance was marginal (*P* = 0.06). Furthermore, plasma CRP concentrations were measured in the four subgroups because IL-1α, IL-6, and TNF-α primarily regulate the production of CRP by the liver. As expected, a significant increase was found for plasma CRP concentrations in overweight and obese groups with a BMI higher than 25 kg/m^2^ compared to a subgroup with a BMI lower than 25 kg/m^2^ (Table 3).

**Table 3:** Plasma IL-1α and CRP concentrations in the four BMI subgroups.

BMI (kg/m^2^)	IL-1α (pg/ml)	*P* a	CRP (ng/ml)	*P* a
<25	7.65±0.55	-	0.70±0.54	-
25–26	7.02±0.59	0.174	3.04±0.07	<0.001
27–29	8.39±1.10	0.064	6.17±1.05	<0.001
≥30	7.86±0.62	0.454	6.71±2.10	<0.001

BMI, body mass index. Values are means ± SD.

a By *t* test; women with BMI <25 kg/m^2^ were the reference in this analysis.

### Transcriptional Activity of IL-1α Polymorphism in Mouse Adipocytes

**Figure 1:**
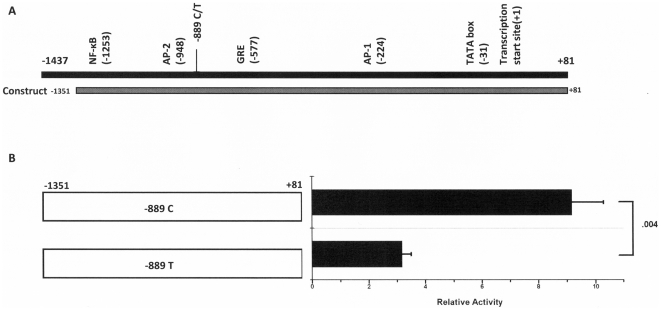
Effect of IL-1α CC or TT at position –889 on luciferase reporter activity. (**A**) Schematic representation of the 5′-flanking region of the IL-1α gene showing the major transcription start site and the putative transcription factor binding sites. NF-κB = nuclear factor-κB; AP = activator protein; GRE = glucocorticoid response element. (**B**) The reporter plasmids containing a fragment of 1,432 bp covering the IL-1α 5′-flanking (nucleotide −1351 to +81) was transfected into 3T3-L1 cells and measured after 48 h. For an assessment of the transfection efficiency, cells were co-transfected with pSV-β-gal, and the luciferase activity was expressed relative to the pGL3-Basic vector and normalized to the β-galactosidase activity. Data are the mean ± SE of the results from three or more experiments conducted in triplicate.

To examine whether the –889C/T polymorphism in the regulatory region of the IL-1α gene is important for transcriptional activity, transcription levels from three reporter gene constructs were compared in 3T3-L1 adipocytes. The first construct was a negative control that contained a promoter-less luciferase reporter gene. The second and third constructs contained a 1,432 bp fragment covering the IL-1α 5′-flanking (nucleotide −1351 to +81), where one construct had a C nucleotide at position –889 of this fragment, and the other construct contained a T nucleotide at position –889 (Fig. 1A). The CC construct produced a 2.9-fold higher level of luciferase activity than the TT construct (*P* = 0.004) (Fig. 1B).

### IL-1α Levels in Obese Mice and Exogenous IL-1α Administration

**Figure 2:**
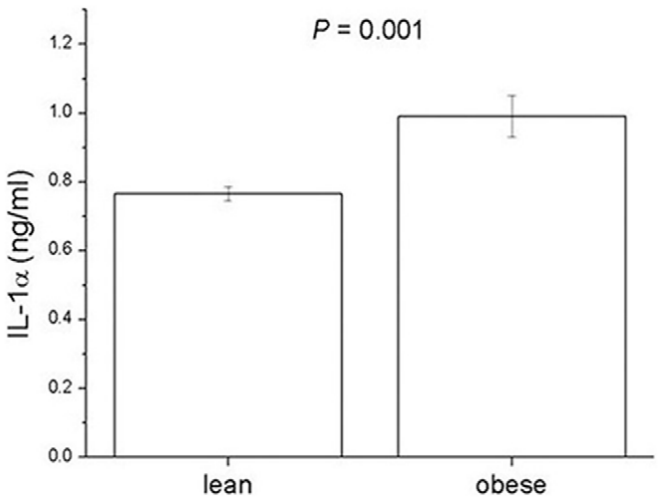
Levels of serum IL-1α in high-fat diet-induced obese mouse model. IL-1α levels were determined by ELISA method. Experiments were performed in duplicate, and values represent means ± S.E.M. (n = 10).

**Figure 3:**
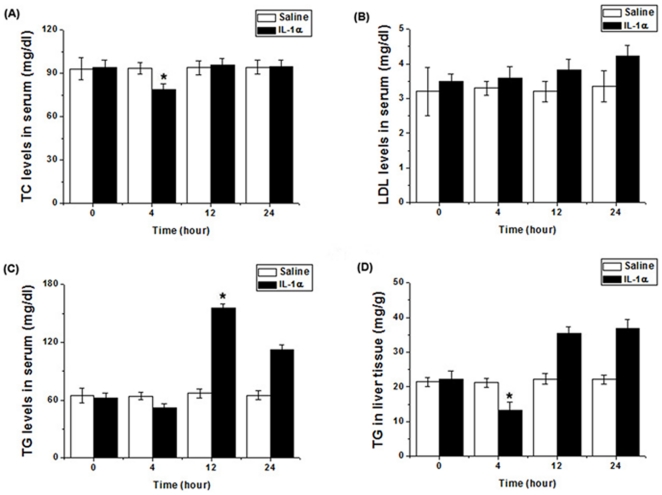
Effect of IL-1α administration on lipids levels. (**A–C**) Serum total cholesterol (TC), triglyceride (TG), and LDL levels in IL-1α administered mice. IL-1α (10 µg/kg body weight) or vehicle (normal saline) was administered IP respectively. At 0, 4, 12, and 24 hours after IL-1α or vehicle injection, blood samples were collected and levels of TC, TG, LDL cholesterol were determined using the colorimetric enzymatic method and an autoanalyzer. (**D**) Liver triglyceride content in IL-1α administered mice. IL-1α (10 µg/kg body weight) or vehicle (normal saline) was administered IP respectively. At 0, 4, 12, and 24 hours after IL-1α or vehicle injection, livers were collected and lipids were extracted from the livers. Levels of triglyceride were determined using the colorimetric enzymatic method and an autoanalyzer. Values represent means ± S.E.M. (n = 6). *P<0.05 as compared to the vehicle injection group.

To examine whether IL-1α is correlated with obesity, the levels from serum of HFD-induced obese mice were determined. IL-1α levels were higher in obese mice than in lean mice (*P* = 0.001) (Fig. 2). Also, we wished to test the effects of exogenous IL-1α administration to mice on lipids levels. IL-1α (10 µg/kg body weight) or vehicle (normal saline) was administered IP respectively. At 0, 4, 12, and 24 hours after IL-1α or vehicle injection, blood levels of total cholesterol (TC), triglyceride (TG), low-density lipoprotein (LDL) cholesterol, and TG from the livers were measured (Fig. 3). At 12 hours after IL-1α injection, the TG level in serum was significantly increased (Fig. 3C).

### IL-1α Expression in Adipose Tissue of Obese Mice

**Figure 4:**
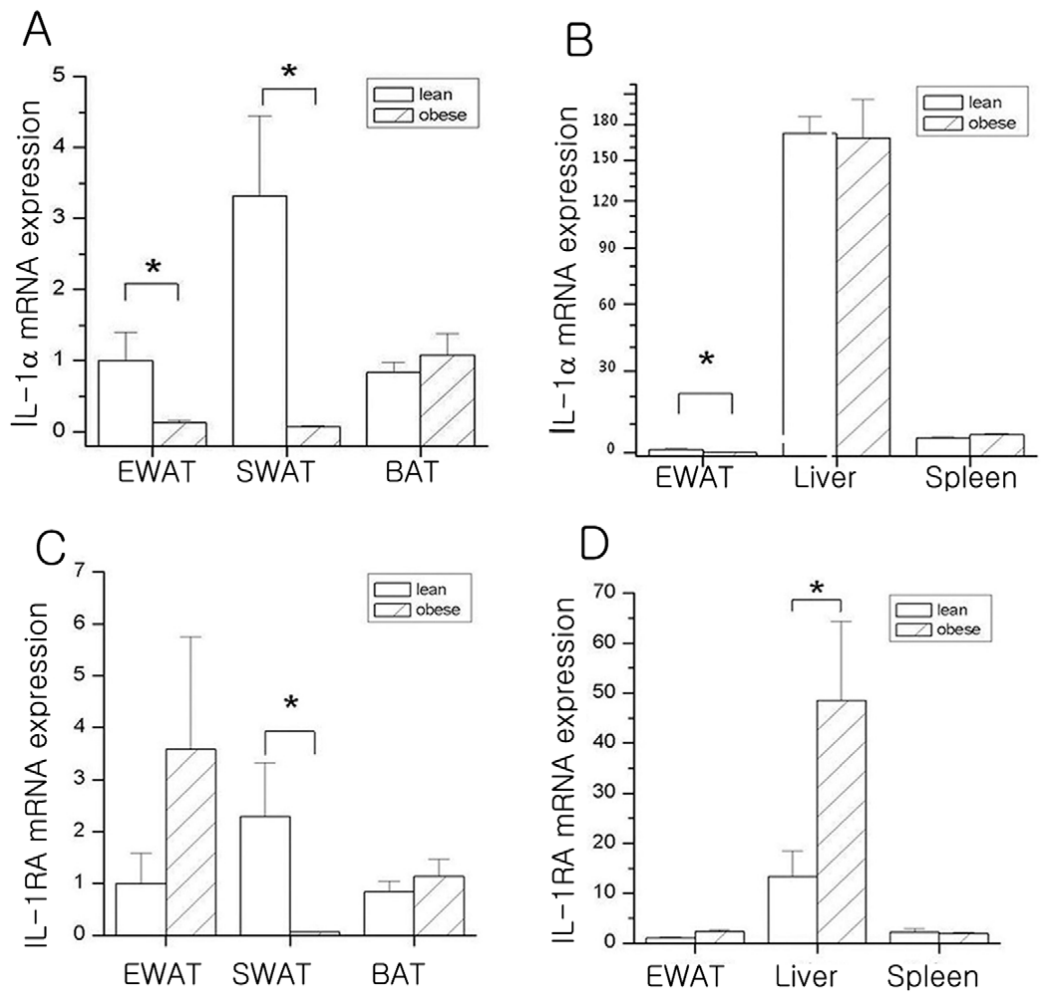
Expression of IL-1α and IL-1Ra in adipose tissue from high-fat diet-induced obese mouse models. Levels of IL-1α mRNA (**A** and **B**) and IL-1 Ra mRNA (**C** and **D**) were determined by quantitative real-time PCR. Experiments were performed in triplicate and values represent means ± S.E.M. (n = 3). IL-1α and IL-1Ra expression levels were normalized to HPRT levels. EWAT: Epididymal White Adipose Tissue; SWAT: Subcutaneous White Adipose Tissue; BAT: Brown Adipose Tissue.

Because IL-1α is elevated in obese mice serum and affects lipid content, we next wished to characterize where the IL-1α is mainly expressed and whether the IL-1α was differentially expressed in lean and HFD-induced obese mice. We first assessed the tissue specific expression pattern of IL-1α by means of real-time quantitative PCR. Although IL-1α mRNA was expressed in epididymal and subcutaneous white adipose tissue (EWAT and SWAT, respectively), as well as brown adipose tissue (BAT), it was mainly expressed in the liver and spleen (Fig. 4A and 4B). When compared to lean mice, EWAT and SWAT from obese mice expressed lower amounts of IL-1α (Fig. 4A). Because IL-1 Ra is a natural antagonist that is specific to IL-1 [23], the presence of IL-1Ra mRNA was measured in the tissues of lean and HFD-induced obese mice. IL-1Ra transcript was expressed in the liver, EWAT, SWAT, BAT, and spleen (Fig. 4C and 4D). In contrast to IL-1α, IL-1Ra was significantly higher in the livers of obese mice as compared to lean mice (Fig. 4D), but it was lower in SWAT of obese mice as compared to lean mice (Fig. 4C).

### Adipocyte Differentiation

**Figure 5:**
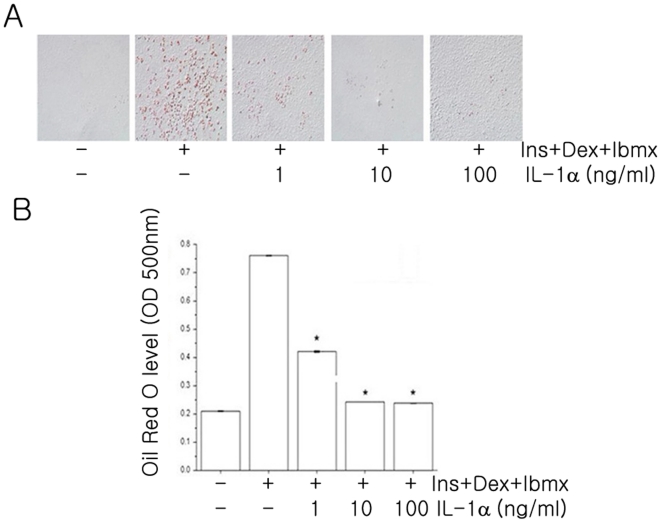
IL-1α inhibits differentiation of preadipocytes. (**A**) The differentiating 3T3-L1 cells were treated with IL-1α at various concentrations for 8 days. Intracellular lipids accumulation was monitored by Oil Red O staining. After staining, dishes were photographed and quantified. (**B**) To estimate the extent of adipose conversion, isopropanol was added to the plate. Optical density was measured at 500 nm using an automated microplate ELISA reader. Values are mean ± S.E.M. of three independent experiments. *P<0.05 compared with untreated and differentiated cells. Ins: Insulin; Dex: Dexamethasone; Ibmx: 3-isobutyl-1-methylxanthine.

Since IL-1α is correlated with obesity in the in vivo study, we wished to determine if IL-1α affected the differentiation process of adipocytes. The differentiating 3T3-L1 cells were cultured in the absence or 1 ng/ml, 10 ng/ml, and 100 ng/ml of IL-1α for 8 days. At day 8, the differentiation was terminated and cells were detected by Oil Red O staining. Untreated differentiated cells had many lipid droplets indicating lipid accumulation. However, lipid accumulation was inhibited by IL-1α treatment in a dose dependent manner (Fig. 5A). This observation was further supported by the quantitative analysis of neutral lipid content by measuring the absorbance at 500 nm (Fig. 5B). Untreated differentiated 3T3L1 cells showed higher levels of lipid staining with the Oil Red O dye, which was significantly reduced by higher doses of IL-1α (10 ng/ml and 100 ng/ml) treatment (Fig. 5B).

**Figure 6:**
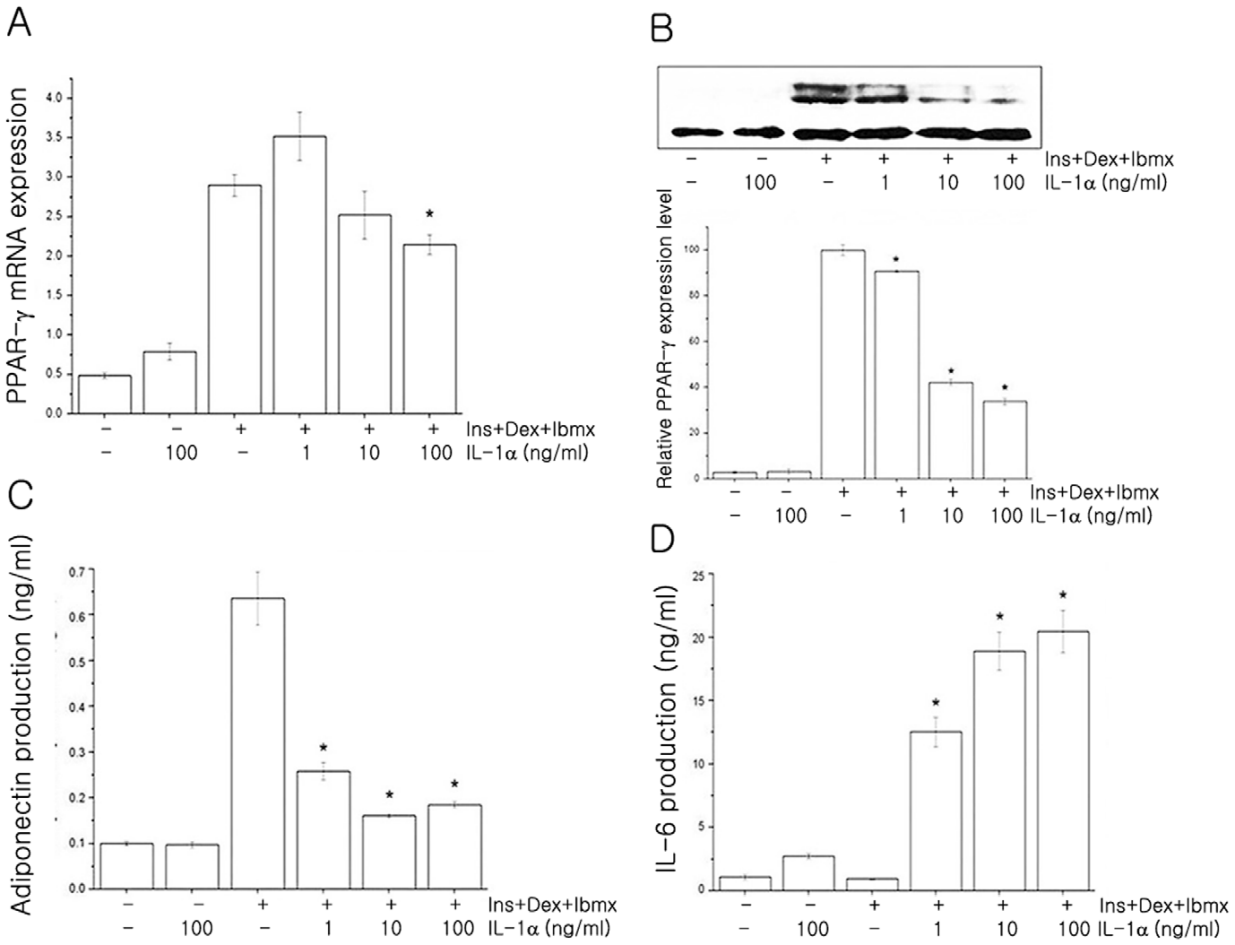
IL-1α inhibits PPAR-γ and adiponectin (but not IL-6) in 3T3-L1 cells. The differentiating 3T3-L1 cells were cultured in the absence or 1 ng/ml, 10 ng/ml, and 100 ng/ml of IL-1α for 8 days. (**A**) Levels of PPAR-γ mRNA were determined by quantitative real-time PCR. PPAR-γ expression levels were normalized to HPRT levels. Experiments were performed in triplicate, and values represent means ± S.E.M. (**B**) The protein expression of PPAR-γ was examined by western blot analysis. Protein levels were quantified by densitometry. Values are mean ± S.E.M. of three independent experiments and are expressed as a percentage relative to the control (100%). (**C**) Adiponectin and (**D**) IL-6 productions were examined by ELISA method. Experiments were performed in duplicate and values are mean ± S.E.M. of three independent experiments. *P<0.05 compared with untreated and differentiated cells. Ins: Insulin; Dex: Dexamethasone; Ibmx: 3-isobutyl-1-methylxanthine.

To identify the mechanism of the inhibition of adipocyte differentiation by IL-1α, the expression of peroxisome proliferator-activated receptor gamma (PPAR-γ), a key transcriptional factor for adipocytes differentiation, was examined. The mRNA and protein expressions of PPAR-γ were significantly inhibited by IL-1α treatment in 3T3-L1 differentiating adipocytes in a dose dependent manner (Fig. 6A and 6B). We further examined the effect of IL-1α on adiponectin and IL-6 production in 3T3-L1 differentiating adipocytes. Adiponectin secretion was suppressed by IL-1α treatment of 1 ng/ml, 10 ng/ml, and 100 ng/ml (Fig. 6C). In contrast, IL-6 production was increased by IL-1α treatment in a dose dependent manner (Fig. 6D).

## Discussion

In this study, we found that both IL-1α polymorphisms C−889T (rs1800587) and G+4845T (rs17561) were associated with an increase in BMI in obese healthy women. In addition, we demonstrated that the polymorphism of rs1800587 affected the transcriptional activity of IL-1α in pre-adipocyte 3T3-L1 cells. We extended these observations in vivo to an HFD-induced obese mouse model and in vitro to pre-adipocyte 3T3-L1 cells. Our findings imply IL-1α may have a critical function in the development of obesity in humans.

In a previous study, we reported an association between the polymorphism in the IL-1β gene and obesity in women. A significant decrease was found for the IL-1β T allele in an overweight group compared to a lean group [19]. In this study, an apparent association between the IL-1α polymorphisms (rs1800587 and rs17561) and BMI in women was found. Additionally, to investigate whether the observed association has any physiological (pathophysiological) relevance, the plasma IL-1α levels were measured in the four BMI subgroups. As expected, circulating IL-1α concentrations tended to be higher in the overweight groups than in the lean group, but not significantly so. We also measured the plasma CRP concentrations in the four groups. CRP is a primitive acute-phase inflammatory protein that is released in response to acute injury, infection, or other inflammatory stimuli, such as hypersensitivity reactions, inflammatory diseases, allograft rejection, malignancy, necrosis, and trauma [24]. Synthesis of CRP occurs in hepatocytes and is regulated primarily by IL-1α, as well as IL-6 and TNF-α [25], [26]. CRP levels are also reported to associate positively with BMI, suggesting that CRP is a useful biomarker for obesity-linked chronic inflammatory states [27]–[29]. As expected, the CRP levels were significantly higher in the overweight groups than in the lean group in this study. However, no association between the genotype of IL-1α -889C/T and the levels of plasma CRP was found (data not shown).

The mechanism by which IL-1α gene polymorphisms influence BMI is unknown. Previous studies have shown that IL-1α production by cultured peripheral blood mononuclear cells in an obese group was significantly higher than in a control group [12] and that healthy non-obese donors with the IL-1α –889TT genotype had increased levels of IL-1α production [16]. Wei *et al.* [30] also reported that C/T conversion increases the activity of the IL-1α promoter in the human astrocyte cell line. Additionally, several studies have reported that chronic inflammatory diseases including rheumatoid arthritis, Alzheimer disease, and periodontitis were associated with the T allele of the IL-1α –889C/T polymorphism [31]–[34]. Thus, it was expected that the T allele carriers might show a higher BMI. However, contrary to all expectations, this study showed an association between the T allele and a lower BMI value. The possible explanations for these discrepancies are not clear. Any effect of this polymorphism on obesity could be influenced by interactions with the ethnic background and other genetic or environmental factors that influence the study population. In addition, to determine the functional significance of the C/T variant in adipocytes, transcription levels from reporter gene constructs containing the –889C/T polymorphism in the IL-1α regulatory region were compared in this study. In vitro studies comparing the –889C and –889T promoters demonstrated decreased transcription from the –889T promoter in 3T3L-1 adipocytes.

To examine whether the IL-1α is correlated with obesity in vivo, we measured the levels of IL-1α from serum of HFD-induced obese mice. As shown in Fig. 2, IL-1α levels were higher in HFD-induced obese mice than in lean mice. We next examined the effects of IP administration of IL-1α to mice. We showed that IP administration of IL-1α to mice increased blood levels of TG at 12 hours after injection. Although there is little research on the effects of administration of IL-1α, Janik et al. [35] reported that IV administration of IL-1α to patients with cancer stimulates leptin production in a dose dependent fashion.

There have been no reports on the effects of obesity on the adipose tissue content of IL-1α. To determine where IL-1α is mainly expressed and whether IL-1α was differentially expressed in lean and obese mice, we measured the expression levels of IL-1α in WAT adipose tissue. As shown in Figs. 4A and 4B, IL-1α mRNA was mainly expressed in the liver and spleen, as well as in EWAT, SWAT, and BAT. When compared to that of lean mice, the EWAT and SWAT from obese mice expressed lower amounts of IL-1α. We also observed that the transcript of IL-1 Ra, a natural antagonist that is specific to IL-1, was expressed in the liver, EWAT, SWAT, BAT, and spleen (Figs. 4C and 4D). In contrast to IL-1α, IL-1Ra was significantly higher in the livers of obese mice than in those of lean mice (Fig. 4D). These data are consistent with work by Juge-Aubry et al. [36], showing that when compared to the tissues of lean mice, most tissues from obese animals expressed higher amounts of IL-1Ra, with a 37- and 5-fold increase in EWAT and inguinal WAT (IWAT) from obese mice, respectively. They also showed that in contrast with IL-1Ra, IL-1β mRNA in IWAT was lower in obese animals than in lean mice. Based on these findings, it may be explained by the auto- and paracrine effects of the increased IL-1Ra∶IL-1α ratio.

We next tested whether IL-1α affects the differentiation process of preadipocytes. In 3T3-L1 differentiating adipocytes, IL-1α inhibited the differentiation programme, as shown by the decreased levels of PPAR-γ (Fig. 5 and Fig. 6). In addition, IL-1α treatment inhibited adiponectin secretion but enhanced IL-6 production in a dose dependent manner (Fig. 6). Mrácek et al. [37] reported that IL-1β and LPS (but not IL-6) inhibited differentiation and downregulated PPAR-γ in brown adipocytes. The circulating adiponectin level is an index of insulin sensitivity. It has been shown that IL-1β strongly attenuated adiponectin expression and secretion in both differentiating 3T3-F442A cells and fully differentiated 3T3-L1 adipocytes [38]. Uno et al. [39] reported that long-term IL-1α treatment enhanced IL-6 production in 3T3-L1 adipocytes. IL-1α and IL-1β bind to the same receptors and exert similar biological activities via the activation of similar signaling pathways [40]. However, IL-1α and IL-1β differ dramatically in the sub-cellular compartments in which they are active [41]–[43]. Compared with a number of reports on the role of IL-1β as an endogenous mediator of insulin resistance, studies on the relationship between IL-1α and insulin signaling and obesity are rare.

The role of IL-1α in obesity has not been studied yet, and there are no studies that examine the effect of IL-1α in both in vivo and in vitro. In addition, there are no studies that perform the functional analyses of specific genes of interest resulting from the study of human polymorphism. To delineate the specific role of IL-1α in obesity, we found an apparent association between IL-1α polymorphisms (rs1800587 and rs17561) and BMI in obese women and the effect of the IL-1α variant on transcriptional activity. We further studied this observation in vivo with an HFD-induced obese mouse model and in vitro with 3T3-L1 adipocytes. In the obese mouse model, IL-1α levels were higher in obese mice than in lean mice. In agreement with our results, the work in humans demonstrated that the plasma concentrations of IL-1α and IL-1β were significantly higher in obese women than in non-obese subjects, and the IL-1α levels were correlated with the fat mass% [11]. In addition, triglyceride was increased 12 hours after exogenous IL-1α administration. Paradoxically, IL-1α treatment attenuated the differentiation of preadipocytes through suppression of PPAR-γ and lipid accumulation. A hypothesis to reconcile this unexpected observation is that IL-1α is a homeostatic regulator that opposes excess positive energy balance, wherein elevated IL-1α levels in obesity reflect inadequate compensation, analogous to what occurs with the adipokine leptin. Meanwhile, IL-1α treatment increased IL-6 production. Elevated levels of cytokine could prevent differentiation of preadipocytes to mature cells and simultaneously promote their ability to produce IL-6, which could further contribute to the systemic levels of the cytokine. Our findings imply IL-1α may have a critical function in the development of obesity in humans. We also suggest that further studies should attempt to identify the detailed mechanism for how IL-1alpha-mediated inhibition of adipocyte differentiation and lipid accumulation would contribute to obesity.

## Supporting Information

Table S1 **Frequencies for IL-1α genotypes according to PBF in females.**
(DOCX)

Table S2 **Frequencies for IL-1α genotypes according to WHR in females.**
(DOCX)
