# Supplementary material for: Functional Polymorphism of IL-1 Alpha and Its Potential Role in Obesity in Humans and Mice
Source: PLoS One. 2011 Dec 27;6(12):e29524. doi: 10.1371/journal.pone.0029524 (PMC3246492; doi:10.1371/journal.pone.0029524)
Supplement: Table S1 — Frequencies for IL-1α genotypes according to PBF in females. (DOCX) [file pone.0029524.s001.docx]

**Table S1.** **Frequencies for IL-1α genotypes according to PBF in females (*n* = 260)**

|  |  | PBF (%) | | | *P* ^a^ |
| --- | --- | --- | --- | --- | --- |
|  |  | < 33,  n (%) | 33 - 40, n (%) | ≥ 40,n (%) |  |
| IL-1α C-889T  (rs1800587) | CC | 35 (83.3) | 121 (78.1) | 56 (88.9) | 0.361 |
|  | CT | 7 (16.7) | 32 (20.6) | 7 (11.1) |  |
|  | TT | 0 (0) | 2 (1.3) | 0 (0) |  |
| IL-1α G+4845T  rs17561 | GG | 39 (84.8) | 115 (76.7) | 57 (89.1) | 0.223 |
|  | GT | 7 (15.2) | 33 (22.0) | 7 (10.9) |  |
|  | TT | 0 (0) | 2 (1.3) | 0 (0) |  |
| rs1800587: rs17561 haplotype | | | | | |
| o copies T:T | | 39 (84.8) | 116 (76.8) | 56 (88.9) | 0.095 |
| 1 or 2 copies T:T | | 7 (15.2) | 35 (23.2) | 7 (11.1) |  |

PBF, percentage of body fat. ^a^By χ^2^ test (two-sided).
