# Supplementary material for: Functional Polymorphism of IL-1 Alpha and Its Potential Role in Obesity in Humans and Mice
Source: PLoS One. 2011 Dec 27;6(12):e29524. doi: 10.1371/journal.pone.0029524 (PMC3246492; doi:10.1371/journal.pone.0029524)
Supplement: Table S2 — Frequencies for IL-1α genotypes according to WHR in females. (DOCX) [file pone.0029524.s002.docx]

**Table S2.** **Frequencies for IL-1α genotypes according to WHR in females (*n* = 260)**

|  |  | WHR | | | *P* ^a^ |
| --- | --- | --- | --- | --- | --- |
|  |  | ~ .85,  n (%) | .86 - .90, n (%) | .91 ~,n (%) |  |
| IL-1α C-889T  (rs1800587) | CC | 32 (84.2) | 59 (71.1) | 121 (87.1) | 0.021 |
|  | CT | 5 (13.2) | 23 (27.7) | 18 (12.9) |  |
|  | TT | 1 (2.6) | 1 (1.2) | 0 (0) |  |
| IL-1α G+4845T  rs17561 | GG | 32 (84.2) | 58 (69.9) | 122 (87.8) | 0.008 |
|  | GT | 5 (13.2) | 24 (28.9) | 17 (12.2) |  |
|  | TT | 1 (2.6) | 1 (1.2) | 0 (0) |  |
| rs1800587: rs17561 haplotype | | | | | |
| o copies T:T | | 32 (84.2) | 58 (69.9) | 121 (87.1) | 0.006 |
| 1 or 2 copies T:T | | 6 (15.8) | 25 (30.1) | 18 (12.9) |  |

WHR, ratio of waist-to-hip circumstance. ^a^By χ^2^ test (two-sided).
